# Do Diagnosis Delays Impact Receipt of Test Results? Evidence from the HIV Early Infant Diagnosis Program in Uganda

**DOI:** 10.1371/journal.pone.0078891

**Published:** 2013-11-01

**Authors:** Melissa Latigo Mugambi, Sarang Deo, Adeodata Kekitiinwa, Charles Kiyaga, Mendel E. Singer

**Affiliations:** 1 Department of Epidemiology and Biostatistics, Case Western Reserve University, Cleveland, Ohio, United States of America; 2 Indian School of Business, Hyderabad, India; 3 Baylor College of Medicine Bristol Myers Squibb Children's Clinical Center of Excellence at Mulago Hospital, Kampala, Uganda; 4 AIDS Control Program, Ministry of Health, Kampala, Uganda; University of Cape Town, South Africa

## Abstract

**Background:**

There is scant evidence on the association between diagnosis delays and the receipt of test results in HIV Early Infant Diagnosis (EID) programs. We determine the association between diagnosis delays and other health care system and patient factors on result receipt.

**Methods:**

We reviewed 703 infant HIV test records for tests performed between January 2008 and February 2009 at a regional referral hospital and level four health center in Uganda. The main outcome was caregiver receipt of the test result. The primary study variable was turnaround time (time between sample collection and result availability at the health facility). Additional variables included clinic entry point, infant age at sample collection, reported HIV status and receipt of antiretroviral prophylaxis for prevention of mother-to-child transmission. We conducted a pooled analysis in addition to separate analyses for each facility. We estimated the relative risk of result receipt using modified Poisson regression with robust standard errors.

**Results:**

Overall, the median result turnaround time, was 38 days. 59% of caregivers received infant test results. Caregivers were less likely to receive results at turnaround times greater than 49 days compared to 28 days or fewer (ARR = 0.83; 95% CI = 0.70–0.98). Caregivers were more likely to receive results at the PMTCT clinic (ARR = 1.81; 95% CI = 1.40–2.33) and less likely at the pediatric ward (ARR = 0.54; 95% CI = 0.37–0.81) compared to the immunization clinic. At the level four health center, result receipt was half as likely among infants older than 9 months compared to 3 months and younger (ARR= 0.47; 95% CI = 0.25–0.93).

**Conclusion:**

In this study setting, we find evidence that longer turnaround times, clinic entry point and age at sample collection may be associated with receipt of infant HIV test results.

## Introduction

An estimated 370,000 children contracted HIV globally in 2009 with over 90% of infections occurring as a result of HIV transmission from HIV-infected mothers to their infants during pregnancy, delivery or breastfeeding [1,2]. Without access to treatment, about 30% of HIV-infected infants die before 12 months of age [3] and when treatment is delayed, infants have a greater risk of morbidity and mortality[4]. Due to the aggressive nature of HIV/AIDS in perinatally infected infants, early diagnosis of infants is essential to reduce morbidity and mortality. 

In Uganda, it is estimated that 91,000 infants are born to HIV-infected mothers annually and are therefore at risk of contracting HIV[5].  Early infant diagnosis (EID) in Uganda and other resource-limited settings is conducted using virologic testing on dried blood spot (DBS) samples[6]. However, transport of DBS samples from remote facilities to centralized laboratories using a weak logistics infrastructure has resulted in turnaround times that are as long as 4 to 12 weeks[7]. 

Long and variable turnaround times can adversely impact health outcomes through three routes. First, they cause delays in initiating therapy and increase the risk of disease progression and mortality among perinatally infected infants[4]. Second, they may discourage caregivers (typically mothers of the HIV-exposed infants) from returning for test results[8,9]. Third, long turnaround times delay the process of counseling mothers on appropriate breastfeeding guidelines which may increase the risk of postnatal transmission of HIV[9]. 

To our knowledge, no study has measured the association between turnaround time and result receipt. A measure of this association can help to establish the extent to which operational interventions in EID programs aimed at reducing turnaround time can aid in improvement of result receipt; this would enable the assessment of the economic and public health impact of these interventions. Furthermore, only few studies have considered how other population characteristics and health care system factors may be associated with result receipt (e.g. age of the infant at sample collection, whether the mother-infant pair received prevention of mother-to-child transmission (PMTCT) services)[10,11]. Infant age may be associated with result receipt because of the higher propensity of caregivers to seek healthcare services when infants are younger [12-14]. Caregiver-infant pairs receiving PMTCT services may be more likely to return for results because they are more familiar with the testing system and more aware of the importance of testing infants for HIV [15]. Infant HIV status, a proxy of health status, may be associated with result receipt because infants who are HIV-infected may be lost to follow-up as a result of rapid disease progression and subsequent morbidity and mortality. Alternatively, frequent illnesses may result in the caregiver seeking care at the health facility. It is important to understand the factors that are associated with result receipt in order to develop more targeted interventions to ensure that test results are received and appropriate and timely care is delivered to HIV-exposed infants[16].

Therefore, in this study, we evaluated the association between receipt of infant HIV test results and turnaround time and other patient factors using data from two health facilities participating in Uganda’s EID program. 

## Methods

### Study Setting and Site Selection

Uganda’s EID program was established in 2007. During the study period (2008 - 2009) about 30% of HIV-exposed infants were tested and 12% of these infants were found to be positive[5]. An estimated 260 facilities, served by seven regional laboratories, were involved in providing EID services (20% of the total number of health facilities at the time) (Uganda Ministry of Health, unpublished data, 2008). Health facilities in Uganda are classified in order of increasing availability of medical services and catchment population size with level one health centers in the lowest tier, followed by level two, three and four health centers, district hospitals, regional referral hospitals and national referral hospitals. We collected data from two government health facilities participating in Uganda’s EID program:  a regional referral hospital (RRH) in Eastern Uganda serving about 2 million people and an urban level four health center (HC4) facility on the outskirts of Kampala serving about 100,000 people[17]. During the study period, the RRH and the HC4 facility referred their samples to two different regional laboratories. We selected the sites based on ease of access, approval by government officials and medical superintendents and an initial screening process in which we verified whether the facility had a sizeable number of test records (at least 100 records) and whether the facility was reported to have comprehensive data on turnaround times and result receipt. 

HIV-exposed infants were referred for testing from different entry points within a facility (e.g. the immunization clinic, pediatric ward etc.) in order to capture as many infants as possible. Referral was contingent upon the mother testing positive for HIV or the infant presenting with signs and symptoms suggestive of HIV/AIDS. The testing process involved the collection of DBS samples from the infants by health workers at the facility. The samples were then shipped to centralized laboratories via courier postal service. Results, when available, were shipped back to the health facility. Caregivers were typically instructed to collect results 4 weeks after sample collection. Key demographic and testing information recorded by the health workers in the testing register included the infant’s name, sex, sample collection date, point of entry in the clinic, whether the infant or mother received antiretroviral therapy prophylaxis to prevent mother-to-child-transmission of HIV, sample shipment date, result receipt date by the health facility and whether or not the caregiver received the result. Information such as infant name, age and whether the caregiver-infant pair received PMTCT services were ascertained by self-report. In this study, only the HC4 had information on whether the caregiver-infant pair received PMTCT services. This information was not available at the RRH. While the RRH had a specific PMTCT referral clinic, data on whether caregivers from other entry points received PMTCT services was not available. If a caregiver returned for a test result and the result was available, the health worker indicated that the caregiver received the result in the register. Infants testing positive were immediately referred to the ART clinic. Caregivers whose infants tested negative were instructed to return for a retest following cessation of breastfeeding. 

### Study Design

Between November 2009 and December 2009, we conducted a retrospective review of 703 records of infant HIV tests that were performed between January 2008 and February 2009. We excluded records from the analysis in which the infant age at sample collection was greater than 18 months. We excluded records with unknown test results namely those that were lost, indeterminate or required a re-run, records that were sent to facilities other than the primary facility from which data was collected, records with missing result receipt data and records where results were indicated to have not been received and yet after further analysis the infant was recorded as enrolled on treatment. We also excluded records that were not recorded in the laboratory register in chronological order (e.g. records that were noted to have been collected in 2008 but were recorded after records collected in 2009) and records with missing turnaround time data or with dates that did not result in plausible turnaround times. 

### Ethical Approval

Approval for this study was obtained from the University Hospitals Institutional Review Board, Cleveland Ohio, the Mbarara University of Science and Technology Faculty of Medicine Research Ethics Committee and the Uganda National Council for Science and Technology with clearance from the Uganda Ministry of Health. A waiver of informed consent was requested and granted by the Institutional Review Boards because the retrospective review was not more than minimal risk and the study procedure did not involve any personal contact with the infants and their caregivers. A waiver of HIPAA authorization was also requested and granted by the Institutional Review Boards. The study could not be practicably conducted without a waiver given the difficulty in tracking infants and caregivers who may have come from more remote and various areas and who may not have a means of contact. The study adhered to HIPAA's privacy regulations.

### Study Variables

The main outcome in the analysis was a dichotomous variable indicating whether or not the caregiver received the infant HIV test result. If test records indicated that the caregiver did not receive test results at the time of data collection (at least 8 months since sample collection) we assumed that the test result was not received by the caregiver. The primary study variable, a health care system factor, was result turnaround time defined as the time interval between the date of DBS sample collection and the date of test result availability at the health facility. We plotted a graph depicting the proportion of result received by turnaround time to identify turnaround time values in which there was a notable change in the proportion of results received also known as a threshold effect. The threshold effects occurred at turnaround time cutoff values of 28 days and 49 days[18]. The World Health Organization (WHO) also recommends that the test results should be received by caregivers within 4 weeks (28 days)[19]. We therefore categorized turnaround time as follows: less than or equal to 28 days, 29 to 49 days and greater than 49 days.

Additional study variables of interest included in the analysis were population characteristics (age of the infant at sample collection, infant sex, reported infant HIV status, whether the mother-infant pair received PMTCT services during labor or delivery) and a health care system factor (the clinic or ward from which the infant was referred for testing or clinic entry point). A graphical analysis of proportion of result receipt by infant age at DBS sample collection demonstrated threshold effects at 3 months of age and 9 months of age. We therefore categorized infant age at sample collection as follows: less than or equal to 3 months of age, 3 to 9 months of age and greater than 9 months of age. PMTCT data were not available from the RRH and clinic entry point data were not available from the HC4. 

### Statistical Analysis

The caregiver served as the unit of the analysis. We conducted a descriptive analysis of the overall study sample. A Kruskal-Wallis test was used to compare continuous non-normal data by facility and clinic entry point while chi-squared tests were used for categorical data. We conducted a pooled analysis using variables that were consistently recorded across both health facilities. We used modified Poisson regression with robust standard errors in the unadjusted and adjusted analyses to estimate the relative risk of result receipt for each study variable[20,21]. For the pooled model, in addition to the clinic entry points in the RRH, we treated the HC4 as a clinic entry point because based on our interviews with the health care workers, we expected the patient population to be relatively homogeneous given the small size of the facility and because testing was primarily performed at the immunization and post-natal clinics alone. In an additional analysis, we evaluated the overall proportion of missing result receipt data and whether there was any difference between records with and without missing result receipt data based on key study variables. We conducted a sensitivity analysis to determine the impact of the study variables on result receipt when records with missing result receipt data were all classified as either received by the caregiver or not received. We also conducted a sensitivity analysis to determine whether our results were robust to changes in the turnaround time threshold of 28 days to values ranging from 26 to 30 days. Finally, we constructed a boxplot to determine if there were any outliers among the turnaround time values. We repeated the regression analysis to determine whether our results were robust to exclusion of the outliers. Data were analyzed using STATA Software for Windows, version 10.1 (StataCorp, TX, USA). 

## Results

### Study sample characteristics

**Figure 1:**
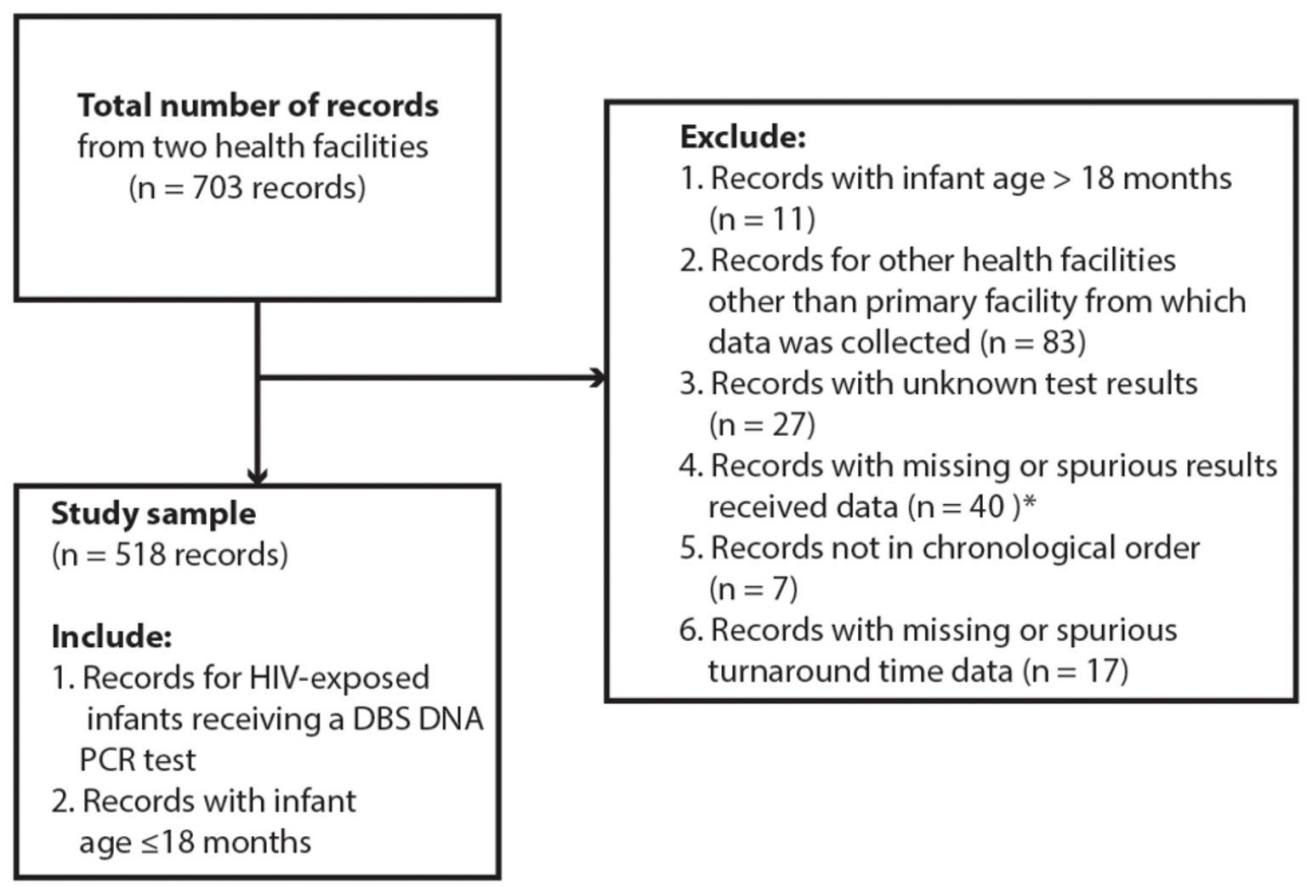
Record selection flowchart. * 32/40 records indicated that result receipt data was missing; 8/40 records indicated that the result was not received but corresponding clinic records showed that the infant was enrolled in treatment.

The process of record selection for analysis is depicted in Figure 1. From our initial sample of 703 records, 518 records from two health facilities were included in the analysis: 131 from the HC4 and 387 from the RRH. Table 1 shows the study sample characteristics. Overall 59% of caregivers received infant test results; 53% at the HC4 and 61% at the RRH (p = 0.085). Overall the median result turnaround time was 38 days (IQR: 25, 54) with a range of 6 to 248 days. The median turnaround time at the HC4 and RRH was 32 days (IQR: 24, 45) and 42 days (oneQR: 26, 66) respectively (p<0.0001). The overall median age at DBS sample collection was 5 months (IQR: 2.5, 9); 3 months (IQR: 1.5, 8) at the HC4 and 6 months (IQR: 3, 10) at the RRH (p<0.0001). Ten per cent of HIV-exposed infants were tested by 6 weeks of age. Eighteen per cent of the test results were positive; 11% (95% CI: 6% - 17%) at the HC4 and 20% (95% CI: 16% - 24%) at the RRH (p = 0.025). Fifty-three per cent of the infants were female and 47% were male. At the HC4, 79% of caregivers reported having received PMTCT services. This information was not available at the RRH. At the RRH infants were referred for testing from the prevention of mother-to-child transmission (PMTCT) referral clinic (32%), pediatric ward (26%), outpatient clinic (16%), immunization clinic (16%) and other clinics (10%) namely the therapeutic feeding clinic, The AIDS Support Organisation (TASO) Uganda and the Baylor Pediatric Infectious Disease Clinic. Table 2 shows the RRH sample characteristics by clinic entry point. Among the clinic entry points, a high proportion of result receipt was observed among caregivers whose infants were referred for testing through the PMTCT referral clinic (95%) while a low proportion of result receipt was observed in the pediatric ward (28%). Infants tended to be significantly younger at sample collection in the PMTCT referral and immunization clinics. The HIV positivity rate was lowest in the PMTCT referral clinic (3%) and highest in the pediatric ward (32%). We found no significant differences in turnaround time and infant sex among the entry points. 

**Table 1:** Characteristics of the Study Sample from the Early Infant Diagnosis Program by Facility.

**Variable**		**Overall (n = 518)**	**Level Four Health Center (n = 131)**	**Regional Referral Hospital (n = 387)**	**p-value**
**Receiving test results (%**)		59	53	61	0.085
**Result turnaround time in days, median (IQR)**		38 (25, 54)	32 (24, 45)	42 (26, 66)	<0.0001
**Age at testing in months, median (IQR**)		5 (2.5, 9)	3 (1.5, 8)	6 (3, 10)	<0.0001
**HIV positive infants (%)**		18	11	20	0.025
**Female (%)**		53	50	55	0.396
**Receiving PMTCT services (%)†**		N/A	75.8		N/A
**Clinic Entry Point (%)‡**	Immunization	N/A	N/A	16	N/A
	Outpatient clinic			16	
	Pediatric ward			26	
	PMTCT referral			32	
	Other			10	

^†^ PMTCT – prevention of mother-to-child transmission services – caregiver received prevention prophylaxis and counseling; this information was not available at the regional referral hospital

^‡^ Clinic entry point data was not available at the level four health center; however infants were largely from the postnatal and immunization clinics; other clinic refers to the therapeutic feeding clinic, The AIDS Support Organisation (TASO) Uganda and the Baylor Pediatric Infectious Disease Clinic.

**Table 2:** Characteristics of the Study Sample from the Early Infant Diagnosis Program by Clinic Entry Point.

**Variable**	**Immunization clinic (n = 60)**	**Outpatient clinic (n = 63)**	**Pediatric Ward (n = 100)**	**PMTCT Referral (n = 122)**	**Other† (n=37)**	**p value**
**Receiving test results (%)**	52	52	28	95	70	<0.0001
**Result turnaround time in days, median (IQR)**	37 (26,66)	38 (25, 80)	42 (27, 54)	44.5 (26, 56)	33 (25, 42)	0.18
**Age at testing in months, median (IQR)**	4 (2.5, 8)	8 (4,12)	6.5 (4, 12)	3.3 (2.5,6)	8.5 (7, 11.5)	0.0001
**HIV positive infants (%)**	17	22	32	3	18	<0.0001
**Female (%)**	63	53	56	52	46	0.48

^†^ Other clinic refers to the therapeutic feeding clinic, The AIDS Support Organisation (TASO) Uganda and the Baylor Pediatric Infectious Disease Clinic.

### Pooled Analysis

Table 3 shows the unadjusted and the adjusted analysis for result receipt using the pooled data. Turnaround time was associated with result receipt in the adjusted analysis; caregivers were less likely to receive test results when turnaround time for test results exceeded 49 days compared to turnaround times of 28 days or fewer (ARR = 0.83; 95% CI = 0.70 - 0.98; n = 513). In the unadjusted analysis, caregivers were less likely to receive test results if their infants’ age at sample collection was greater than 9 months compared to 3 months or fewer (RR = 0.70; 95% CI = 0.56 - 0.87; n = 518) however age was not associated with result receipt in the adjusted analysis. Compared to the immunization clinic, caregivers whose infants were referred for testing through the pediatric ward were less likely to receive test results (ARR = 0.54; 95% CI = 0.37 - 0.81; n = 513) whereas caregivers whose infants were referred for testing through the PMTCT referral clinic were more likely to receive test results (ARR = 1.81; 95% CI = 1.40 - 2.33; n = 513). The association between reported infant HIV status and result receipt showed borderline significance in the unadjusted analysis with caregivers of positive infants being less likely to receive results (RR = 0.81; 95% CI = 0.65 - 1.01; n = 518). However, infant HIV status was not associated with result receipt in the adjusted analysis. 

**Table 3:** Unadjusted and Adjusted Analysis of Result Receipt at the Level Four Health Center and Regional Referral Hospital.

**Variable**		**Unadjusted Relative Risk**	**95% CI**	**Adjusted Relative Risk**	**95% CI**
**Result turnaround time**	≤ 28 days	1.00		1.00	
	29 - 49 days	0.99	0.84 - 1.16	0.96	0.83 - 1.11
	> 49 days	0.90	0.74 - 1.09	0.83	0.70 - 0.98
**Age**	≤3 months	1.00		1.00	
	3 - 9 months	0.95	0.82 - 1.11	1.01	0.88 - 1.16
	> 9 months	0.70	0.56 - 0.87	0.87	0.70 - 1.09
**Result**	Negative	1.00		1.00	
	Positive	0.81	0.65 - 1.01	1.01	0.79 - 1.30
**Clinic Entry Point**	Immunization	1.00			
	Outpatient clinic	1.01	0.72 - 1.42	1.03	0.73 - 1.45
	Pediatric ward	0.54	0.36 - 0.81	0.54	0.37 - 0.81
	PMTCT referral	1.84	1.44 - 2.36	1.81	1.40 - 2.33
	Other‡	1.36	0.99 - 1.88	1.33	0.95 - 1.85
	HC4	1.02	0.76 - 1.37	0.98	0.73 - 1.32

HC4: level four health center; the HC4 was modeled as an additional clinic entry point in the pooled analysis

^‡^ Other clinic refers to the therapeutic feeding clinic, The AIDS Support Organisation (TASO) Uganda and the Baylor Pediatric Infectious Disease Clinic.

### Level Four Health Center Analysis

Table 4 shows the unadjusted and adjusted analysis for result receipt at the HC4. Turnaround time was not associated with result receipt at the HC4. Caregivers were less likely to receive test results if their infants’ age at sample collection was greater than 9 months compared to 3 months or fewer at the HC4 in the adjusted analysis (ARR= 0.47; 95% CI = 0.25 - 0.93; n=131). Infant HIV status and the receipt of PMTCT services were not associated with result receipt. 

**Table 4:** Unadjusted and adjusted analysis for result receipt at the Level Four Health Center.

**Variable**		**Unadjusted Relative Risk**	**95% CI**	**Adjusted Relative Risk**	**95% CI**
**Result turnaround time**	≤ 28 days	1.00		1.00	
	29 - 49 days	0.84	0.56 - 1.20	0.90	0.64 - 1.26
	> 49 days	0.81	0.48 - 1.38	0.89	0.52 - 1.51
**Age**	≤ 3 months	1.00		1.00	
	3 - 9 months	0.75	0.50 - 1.10	0.75	0.51 - 1.12
	> 9 months	0.46	0.24 - 0.88	0.47	0.25 - 0.93
**Result**	Negative	1.00		1.00	
	Positive	0.60	0.29 - 1.26	0.62	0.31 - 1.26
**PMTCT services†**	No	1.00		1.00	
	Yes	1.17	0.76 - 1.81	0.97	0.64 - 1.47

^†^ PMTCT – prevention of mother-to-child transmission services – caregiver received prevention prophylaxis and counseling; this information was not available at the regional referral hospital

### Regional Referral Hospital Analysis

Table 5 shows the unadjusted and adjusted analysis for result receipt at the RRH. Turnaround time was not associated with result receipt; however, the relative risk of result receipt was comparable to that observed in the pooled analysis when turnaround time for test results exceeded 49 days compared to turnaround times of 28 days or fewer (ARR= 0.85; 95% CI = 0.71 - 1.01; n=382). Caregivers were less likely to receive test results if their infants’ age at sample collection was greater than 9 months compared to 3 months or fewer (RR= 0.75; 95% CI = 0.59 - 0.95; n=387) in the unadjusted analysis. Age at sample collection was not associated with result receipt in the adjusted analysis. Compared to the immunization clinic, caregivers whose infants were referred for testing through the pediatric ward were less likely to receive test results (ARR = 0.52; 95% CI = 0.35 - 0.77; n = 382) whereas caregivers whose infants were referred for testing through the PMTCT referral clinic were more likely to receive test results (ARR = 1.88; 95% CI = 1.46 - 2.44; n = 382). Infant HIV status was not associated with result receipt. 

**Table 5:** Unadjusted and Adjusted Analysis for Result Receipt at the Regional Referral Hospital.

**Variable**		**Unadjusted Relative Risk**	**95% CI**	**Adjusted Relative Risk**	**95% CI**
**Result turnaround time**	≤ 28 days	1.00		1.00	
	29 - 49 days	1.04	0.87 - 1.25	0.98	0.84 - 1.16
	> 49 days	0.90	0.73 - 1.11	0.85	0.71 - 1.01
**Age**	≤ 3 months	1.00		1.00	
	3 - 9 months	0.98	0.83 - 1.17	1.13	0.98 - 1.31
	> 9 months	0.75	0.59 - 0.95	1.06	0.84 - 1.35
**Result**	Negative	1.00		1.00	
	Positive	0.83	0.66 - 1.04	1.12	0.86 - 1.47
**Clinic Entry Point‡**	Immunization	1.00		1.00	
	Outpatient clinic	1.01	0.72 - 1.42	1.00	0.72 - 1.40
	Pediatric ward	0.54	0.36 - 0.81	0.52	0.35 - 0.77
	PMTCT referral	1.84	1.44 - 2.36	1.88	1.46 - 2.44
	Other‡	1.36	0.99 - 1.88	1.22	0.88 - 1.71

^‡^ Other clinic refers to the therapeutic feeding clinic, The AIDS Support Organisation (TASO) Uganda and the Baylor Pediatric Infectious Disease Clinic.

### Sensitivity Analysis

Overall missing result receipt data accounted for 5.8% of the records and the outpatient clinic had a higher proportion of missing result receipt data (25%) compared to all other entry points (2.4%). The infant age at sample collection among records with missing data (median: 11; IQR: 6, 14; n=32) was significantly higher compared to records without missing data (median: 5; IQR: 2.5, 9; n=518; p = 0.0001). However, our results describing the impact of the study variables on result receipt were robust to classification of missing results as either received by the caregiver or not received. Our results were also robust to changes in the turnaround time threshold of 28 days and to exclusion of the outlying turnaround time values. 

## Discussion

Based on combined data from a regional referral hospital (RRH) and a level four health facility (HC4) in Uganda, caregivers were 17% less likely to receive their infants’ test results when turnaround time for test results exceeded 49 days compared to turnaround times of 28 days or fewer. Compared to the immunization clinic, caregivers whose infants were referred for testing through the PMTCT clinic were 81% more likely to receive test results and 46% less likely to receive test results if their infants were referred through the pediatric ward. At the HC4, infant age at sample collection was also associated with result receipt. Caregivers of infants whose samples were collected at age 9 months or older were 53% less likely to receive results than caregivers of infants whose samples were collected at age 3 months or younger. 

The long turnaround times observed in this study sample may be attributed to logistical challenges such as unavailability of regular transport opportunities at the health facilities, high workload at the laboratory, the practice of processing samples in large batches, and communication failures between postal service centers and health facilities. There are several reasons that might explain the association between turnaround time and result receipt. Lengthy delays beyond the 4-week threshold at which caregivers are instructed to collect results, may lead to caregivers making multiple attempts to collect results and eventually giving up after failure to get the results. Failure to receive results may additionally be exacerbated by the natural attrition of caregivers and infants from EID programs over time[9]. It is important to note that we did not observe a significant association between turnaround time and result receipt at the individual facility level as well as in the unadjusted pooled analysis. However, the relative risk estimates were comparable to those observed in the adjusted pooled analysis. Failure to observe an association may have been due to the lack of power in the individual facility analyses. 

 We found that older age at sample collection resulted in decreased result receipt at the HC4. There are several potential explanations for the association at the HC4. After 9 months of age, health workers may have employed adult rapid HIV tests and used these test results to inform patient care thereby reducing the importance of EID results; adult rapid tests have been shown to be feasible in this age group given the decline in maternal HIV antibodies by this age[19,22,23]. Lower return rates may also be seen given that the immunization schedule is typically completed around 9 months of age per the Uganda Child Health Card therefore decreasing the likelihood of the caregiver returning to the health facility[24]. Alternatively, testing an infant at an earlier age may have been associated with caregivers who demonstrated improved child-health seeking behavior. The failure to observe a relationship between age at sample collection and result receipt at the RRH may be due to the confounding effect of clinic entry point. Caregivers from the PMTCT referral clinic were more likely to receive results and their infants tended to be younger compared to infants from other clinic entry points.

We found that clinic entry point was an important factor associated with result receipt, an indication that operational practices and population characteristics may differ among clinics housed within the same health facility. Higher levels of result receipt among infants from the PMTCT referral clinic may have occurred because these patients were likely to be regular attendees of the clinic, more familiar with the system practices and part of a better patient-tracking system. On the other hand patients attending the pediatric ward may have been referred from more remote areas and discharged prior to result availability leading to lower result receipt levels[25]. Infants in the pediatric ward have also been shown to be sicker and have higher positivity rates in this and other studies; therefore failure to receive results might also be a marker of mortality [25,26]. Our findings are similar to that of another study in Malawi which found that a significantly lower proportion of caregivers in the general pediatric clinic received infant test results compared to caregivers in the immunization clinic[10]. 

Our study has limitations. Firstly, we selected our study sample from two health facilities that represented a small fraction of the then 260 facilities in Uganda’s EID program (Uganda Ministry of Health, unpublished data, 2008). Inclusion of additional facilities posed a data collection challenge because the data had to be collected from paper records stationed in each health facility rather than a centralized location which would have streamlined the data collection process. Moreover, given the nascent stage of the EID program, study variables were not consistently recorded among health facilities. Therefore, our findings may not be representative of other health facilities in Uganda’s EID program. 

Secondly, as a retrospective study, we only had programmatic data that is routinely collected and were unable to include caregiver variables such as number of return attempts or whether the caregiver was on antiretroviral therapy. Thirdly, we used result receipt as our main outcome variable. While a valid EID performance metric, it is an intermediate outcome in the EID cascade and we were unable to predict the impact of turnaround time on infant morbidity and mortality and on loss to follow-up further along the cascade. 

 Finally, since our study, Uganda’s EID program has experienced notable changes including the creation of a consolidated robust EID laboratory and the set-up of a national sample transport network by the Ministry of Health, which has reduced the turnaround time to 7 to 14 days (Uganda Ministry of Health, unpublished data, 2012). While the turnaround time values observed in our study sample may not be representative of the country’s current EID operations, the challenges of turnaround time and result receipt are still evident in the larger sub-Saharan Africa region [7,8,26]. Since the EID program’s inception, infant HIV testing coverage increased from 12.3% of the target population in 2007 to 40.2% in 2011, while the estimated positivity rate decreased from 19.4% in 2007 to 7.4% in 2011[5]. The positivity rate in our study sample is not representative of the entire country because our sample was from an earlier phase of the EID program when sick infants would have been overrepresented at the testing stage. Moreover, a large fraction of our observations, which are from the RRH, are likely to get sicker infants as referrals from other health facilities. However, our study findings suggest that operational interventions designed to reduce turnaround time may be helpful in increasing infant test result receipt among caregivers in EID programs. Additionally, our study confirms a strong association between clinic entry point and result receipt that has previously been observed[10]; our study suggests a potential role for infant age at sample collection in understanding the dynamics of result receipt in EID programs. 

In summary, we found that result receipt was less likely at longer turnaround times, among infants referred for testing through a pediatric ward and among older infants in one health facility. The association between turnaround time and result receipt can be used as a critical input to quantify the effectiveness of operational interventions designed to reduce turnaround time in terms of patient outcomes. This study suggests that interventions which aid in reducing turnaround time such as short message service (SMS) delivery of test results or point-of-care testing are likely to be effective but the clinical impact of improved result receipt remains to be studied. Additionally, more targeted efforts may be required to ensure that results are received among specific populations including caregivers whose infants are referred for testing through the pediatric ward or whose infants are older. Future studies should include a larger number of health facilities as it will provide more statistical power and also help to capture the heterogeneity of the association across facilities. While our study was not designed to understand the underlying reasons for the association between turnaround and result receipt, we acknowledge that caregiver idiosyncrasies in returning for results may influence the association. We therefore encourage further studies that account for caregiver behavior and return patterns in EID programs. 
